# *TGF-βI/FERMT2*/*COL6A1* Reciprocal Loop Drives Tumor-Stroma Crosstalk and Promotes Peritoneal Metastasis in Gastric Cancer

**DOI:** 10.7150/ijbs.119895

**Published:** 2025-09-12

**Authors:** Chao He, Zheng Zhou, Jiayue Ye, Xiangliu Chen, Yan Yang, Xinguang Jin, Quan Zhou, Lisong Teng

**Affiliations:** 1 Department of Surgical Oncology, The First Affiliated Hospital, School of Medicine, Zhejiang University, Hangzhou, China.; 2 Department of Thoracic Surgery, the First Affiliated Hospital, Zhejiang University School of Medicine, Hangzhou, China.; 3 Department of Gastric Surgery, Zhejiang Cancer Hospital, Hangzhou Institute of Medicine (HIM), Chinese Academy of Sciences, Hangzhou, Zhejiang, China.

**Keywords:** gastric cancer, peritoneal metastasis, cancer-associated fibroblasts, tumor microenvironment, exosomes

## Abstract

**Background:** Peritoneal metastasis (PM) is a frequent and fatal progression in advanced gastric cancer (GC), shaped by intricate interactions between tumor cells and the tumor microenvironment. Among these, gastric cancer-associated fibroblasts (GCAFs) are key mediators of tumor progression, yet the molecular regulators underlying tumor-stroma crosstalk remain poorly defined.

**Methods:** We combined bulk and single-cell transcriptomics, functional assays, proteomics, and in vivo models to dissect the role of *FERMT2* in modulating GC-GCAF interactions and its contribution to peritoneal dissemination.

**Results:** *FERMT2* is highly expressed in CAFs and positively correlates with both CAF infiltration and activation in GC. Functionally, *FERMT2* maintains the myofibroblastic phenotype of GCAFs by acting as a competing endogenous RNA (ceRNA) for *ZEB2*, thereby promoting *α-SMA* transcription. *FERMT2* also drives GCAF-derived secretion of transforming growth factor-beta 1 (TGF-β1), which in turn induces *FERMT2* expression in GC cells, enhancing their migration, invasion, and resistance to anoikis. In parallel, tumor-derived *FERMT2* upregulates *COL6A1* and facilitates its transfer to GCAFs via exosomes, amplifying *TGF-β* signaling and reinforcing CAF activation. Intracellular COL6A1 sustains the pro-metastatic phenotype of GCAFs. Together, these interactions constitute a *TGF-β1*/*FERMT2*/*COL6A1* positive feedback loop that fuels tumor-stroma crosstalk and promotes peritoneal dissemination in GC.

**Conclusion:** This study identifies a reciprocal regulatory loop involving *FERMT2*, *TGF-β1*, and *COL6A1*, which promotes tumor-stroma interaction and peritoneal dissemination, suggesting a potential therapeutic target for advanced gastric cancer.

## Introduction

Gastric cancer ranks as the fifth most commonly diagnosed malignancy and the third leading cause of cancer-related mortality worldwide 1. The five-year survival rate for GC remains alarmingly low, primarily due to the challenges of early detection and the limited efficacy of curative resection 2. Despite advances in surgical techniques and adjuvant therapies, the prognosis for patients with advanced GC is still poor 3. Notably, at least 33.9% of GC cases present with distant metastasis at the time of diagnosis 4, and approximately 14% of patients exhibit peritoneal metastasis at the time of initial surgery, a condition associated with a median survival of less than six months 5. These dismal outcomes highlight the urgent need to elucidate the molecular mechanisms underlying PM in GC. Understanding the key drivers and adverse prognostic factors of PM could pave the way for the development of targeted therapies and novel strategies for its prevention and clinical management.

Malignant behaviors in tumors are profoundly influenced by the complex interplay between cancer cells and the tumor microenvironment (TME) 6,7. Tumor biology is therefore dictated not only by the characteristics of neoplastic epithelial cells but also by the regulatory signals from stromal components 8. *FERMT2*, also known as *Kindlin-2* or *PLEKHC1*, is a crucial member of the Kindlin family of focal adhesion proteins and has emerged as a key player in the pathogenesis and progression of various malignancies 9. As a pivotal regulator of integrin signaling, *FERMT2* promotes tumor progression by modulating various cellular components within the TME. In breast cancer, *FERMT2* enhances tumor-associated macrophage (TAM) infiltration by upregulating the expression and secretion of colony-stimulating factor 1 (CSF-1) from tumor cells 10. In pancreatic cancer, *FERMT2* is highly expressed in pancreatic stellate cells, where it drives tumor progression through the release of pro-inflammatory cytokines 11. In oral squamous cell carcinoma (OSCC), *FERMT2* regulates the expression and secretion of extracellular matrix proteins such as SPARC and COL4A1 in CAFs, promoting epithelial-mesenchymal transition (EMT) and M2 macrophage polarization 12. Collectively, these findings underscore *FERMT2* as a critical regulator of TME remodeling across multiple tumor types, highlighting its potential as a therapeutic target for disrupting tumor-stroma crosstalk.

CAFs represent one of the most prominent stromal cell populations in various solid tumors, including GC 13. Within the TME, CAFs play a central role in tumor progression by regulating extracellular matrix (ECM) remodeling, promoting tumor growth and metastasis, facilitating immune evasion, and contributing to therapy resistance 14. Consequently, targeting CAF function to disrupt their bidirectional communication with cancer cells and reverse the fibrotic TME holds considerable promise for improving therapeutic outcomes in GC. Our previous bioinformatic analyses revealed that *FERMT2* is predominantly expressed in stromal cells, especially fibroblasts, across multiple cancer types, with its expression strongly correlating with the infiltration and activation of CAFs within the TME 15. This suggests that *FERMT2* may facilitate GC progression and peritoneal metastasis by regulating CAF-mediated ECM remodeling.

In this study, we aim to systematically elucidate the regulatory role of *FERMT2* in the crosstalk between GC cells and GCAFs, and to investigate the molecular mechanisms by which *FERMT2* influences GCAF-driven peritoneal metastasis in GC.

## Methods

### Isolation of CAFs and NFs

CAFs and normal fibroblasts (NFs) were isolated from tumor and matched adjacent non-tumor tissues of gastric adenocarcinoma patients. All specimens were pathologically confirmed. Tissues were rinsed with 1% Penicillin-Streptomycin solution and minced into 1 mm³ pieces, followed by digestion with 1 mg/mL collagenase type IV at 37°C for 1 hour. The digestion was stopped with DMEM supplemented with 20% FBS. The cell suspension was filtered, centrifuged, and cultured in DMEM with 20% FBS. Fibroblast identity was confirmed by Western blotting and immunofluorescence.

### Cell lines

Two human gastric cancer cell lines (AGS and HGC-27), along with the non-cancerous gastric epithelial cell line GES-1 (to evaluate the role of exosomes or secreted proteins derived from non-cancerous cells in regulating the behavior of GCAFs), were obtained from the Cell Bank of the Chinese Academy of Sciences (Shanghai, China). Cells were maintained in RPMI-1640 medium with 10% FBS at 37°C in a 5% CO₂ incubator. NFs and GCAFs were cultured similarly in DMEM with 10% FBS.

### Transwell assay for migration and invasion

AGS or HGC-27 cells (2 × 10⁴) were seeded in serum-free DMEM with or without Matrigel (Corning, USA diluted 1:8) in the upper chambers of 24-well transwell inserts. FERMT2-knockdown or wild-type GCAFs (1 × 10⁵) were seeded in the lower chambers. After 24 hours, cells on the underside of the membrane were fixed, stained with crystal violet, and counted.

### EdU cell proliferation assay

GCAFs (5 × 10⁴) were seeded in 6-well plates. EdU (20 μM) was added for 2 hours, and cells were fixed, permeabilized, and stained using the Click™ EdU Kit (Beyotime, China). Nuclei were counterstained with Hoechst 33342.

### Wound healing assay

GCAFs (1 × 10⁵) were seeded in 6-well plates. After reaching 90% confluence, a scratch was made, and cells were incubated in serum-free medium. Wound closure was monitored at 0 and 24 hours.

### Analysis of *FERMT2* in the gastric tumor microenvironment

TCGA, TARGET, and GTEx datasets were obtained from UCSC Xena (https://xenabrowser.net). EPIC scores and CAF infiltration levels were calculated from gene expression data using the IOBR R package (v0.99.0). Spearman's rank correlation between *FERMT2* expression and CAF infiltration across cancer types was assessed using the 'corr.test' function in the psych R package (v2.1.6), with P < 0.05 considered statistically significant. In gastric cancer, the expression of FERMT2 in CAFs and other cellular subsets within the tumor microenvironment was analyzed using single-cell RNA-seq data obtained from the TISCH database (http://tisch.comp-genomics.org/home/).

### Single-cell RNA-seq data processing

Single-cell RNA sequencing data from primary gastric cancer lesions (GSE210347) were obtained from the GEO database and analyzed using the Seurat R package (version 4.3.0.1). Cells with fewer than 200 detected genes or over 20% mitochondrial gene content were excluded, and only cells with 200 to 7,000 unique features were retained. Gene expression data were normalized using the LogNormalize method with a scale factor of 10,000. The top 2,000 highly variable genes were identified using the variance-stabilizing transformation method for downstream analysis. Data scaling was performed with regression of mitochondrial gene effects. Batch correction was carried out using the Harmony R package (version 0.1.1), followed by dimensionality reduction with principal component analysis (PCA), uniform manifold approximation and projection (UMAP), and t-distributed stochastic neighbor embedding (t-SNE). Cell clustering was performed using a resolution parameter of 0.5, and cell types were annotated based on canonical marker gene expression. For further analysis, CAFs were selected and stratified into *FERMT2*-positive and *FERMT2*-negative groups based on FERMT2 expression levels.

### Cell-cell communication analysis

Intercellular communication was analyzed using the CellChat R package (version 1.6.1). Preprocessed single-cell RNA-seq data, including normalized gene expression and cell metadata, were used to define distinct cell populations, incorporating *FERMT2* expression classification. Communication probabilities were computed for each cell type pair, and low-abundance interactions were excluded based on minimum cell number thresholds. The resulting interaction networks were visualized using circular plots, illustrating both the number and strength of inferred interactions.

### Pseudotime analysis

Developmental trajectories of CAFs were analyzed using the Monocle3 R package (version 1.3.7). CAFs were extracted from the Seurat object, and a compatible Cell Data Set (CDS) was constructed using the raw count matrix, cell metadata, and gene annotations. Trajectories were constructed using principal component analysis, and pseudotime values were calculated for each cell to infer differentiation paths. Gene expression dynamics were assessed and plotted across pseudotime to identify stage-specific transcriptional changes.

### Gene Set Enrichment Analysis (GSEA)

GSEA was performed using the singleseqgset R package (version 0.1.2.9000) to evaluate pathway-level alterations among distinct CAF populations. CAF subsets were analyzed based on *FERMT2* expression, and enrichment analysis was conducted using Hallmark gene sets from MSigDB via the msigdbr package (version 7.5.1). In parallel, transcriptomic data from TCGA-STAD were analyzed by stratifying samples into high and low *FERMT2* expression groups, and GSEA was conducted to identify CAF-related pathways significantly associated with *FERMT2* expression.

### Immunohistochemistry

Paraffin-embedded sections were incubated at 65°C for 2 hours, then deparaffinized, rehydrated, and subjected to antigen retrieval. Primary antibodies were applied at volumes proportional to tissue size and incubated at 37°C for 1 hour. After washing, HRP-conjugated goat anti-rabbit IgG polymers were added and incubated for 20 minutes at 37°C. DAB substrate was then applied for 2-5 minutes, followed by hematoxylin counterstaining.

### Immunofluorescence

Cells were seeded on confocal dishes 24 hours prior to staining. The next day, cells were fixed with 4% paraformaldehyde for 15 minutes, permeabilized with 0.5% Triton X-100 for 20 minutes, and blocked with 5% BSA for 30 minutes. Primary antibody (1:200) was added and incubated overnight at 4°C. After washing with PBST, cells were incubated with secondary antibody (1:200) for 1 hour at room temperature in the dark. DAPI was added for nuclear staining, followed by a 5-minute incubation. Samples were mounted with antifade reagent.

### Cell culture supernatant non-targeted proteomics sequencing

Cells were seeded in 20 cm culture dishes and cultured in serum-free medium. Upon reaching 80% confluence, the supernatant was collected, centrifuged at 3500 × g for 5 minutes at 4°C to remove debris, and frozen in liquid nitrogen for 5 minutes before storage at -80°C. Sequencing was performed by Ouyi Biological Company (Shanghai, China).

### Dual-luciferase reporter assay

Plasmids for *ZEB2* overexpression, *α-SMA* promoter-driven luciferase reporter, and their respective controls were constructed by Genomeditech (Shanghai, China). GCAFs were co-transfected with 500 ng of *ZEB2* plasmid or empty vector, 500 ng of *α-SMA* reporter plasmid or pGL3-basic, and 25 ng of internal control plasmid (pRL-TK). After 48 hours, cells were lysed, and luciferase activity was measured using the Dual-Luciferase® Reporter Assay Kit (Yeasen Biotech, Shanghai, China) with a SpectraMax i3x microplate reader (Molecular Devices, Austria).

### Western blot assay

Cells were lysed in RIPA buffer (Beyotime, China) containing phosphatase and protease inhibitors. After centrifugation, the supernatants were used for protein analysis. Protein concentrations were determined by BCA assay, and 20 μg of protein were loaded onto SDS-PAGE gels, transferred to PVDF membranes, and incubated with primary antibodies overnight at 4°C. Membranes were then incubated with HRP-conjugated secondary antibodies and developed using an enhanced chemiluminescence kit (Yeasen Biotech, China). A full list of antibodies is provided in Table S1.

### Cell transfection

Small interfering RNAs (siRNAs) and negative controls (NC) targeting specific genes were synthesized by Qingke Biotech (Guangzhou, China). siRNAs were transfected into GCAFs using Polyplus transfection reagent (France). For stable gene silencing, lentiviral vectors encoding short hairpin RNAs (shRNAs) targeting *FERMT2*, which were packaged and purified by Genomeditech (Shanghai, China), were transfected into GCAFs and selected with puromycin for two weeks. siRNA, shRNA and miR-RNA sequences applied in this study were shown in Table S2.

### ELISA assay

TGF-β1 levels in cell culture supernatants were quantified using a Human TGF-β1 ELISA Kit (Boster Biological Technology, Wuhan, China). Samples were acid-activated, neutralized, and then added to a 96-well plate pre-coated with a TGF-β1-specific antibody. Following incubation and biotin-labeled detection antibody addition, avidin-HRP conjugate was added, and color development was achieved using TMB substrate. Absorbance was measured at 450 nm, and concentrations were calculated from a standard curve.

### Exosomes extraction

Exosomes were isolated from cell culture supernatants using the BeyoExo™ Enhanced Exosome Isolation Kit (Beyotime, China). The supernatant was centrifuged at 500 × g for 5 minutes at 4°C to remove cell debris, followed by a 10,000 × g centrifugation for 1 hour to eliminate large vesicles. The supernatant was filtered through a 0.22 μm membrane and concentrated 10-fold using ultrafiltration. For every 1 mL of concentrated supernatant, 190 μL of BeyoExo™ reagent was added, mixed, and incubated overnight at 4°C. The mixture was centrifuged the next day, and the exosome pellet was collected.

### Animal experiment: *in vivo* xenograft model

An intraperitoneal dissemination model was established using 4-week-old female BALB/c nude mice, which were randomly assigned to four groups (n = 5 per group): 1 OE-NC AGS + sh-NC CAFs, 2 OE-NC AGS + sh-*FERMT2* CAFs, 3 OE-*FERMT2* AGS + sh-NC CAFs, and 4 OE-*FERMT2* AGS + sh-*FERMT2* CAFs. Each mouse received an intraperitoneal injection of a mixture containing 5 × 10⁶ AGS cells and 5 × 10⁶ GCAFs (a total of 1 × 10⁷ cells), suspended in 200 μL of serum-free DMEM and Matrigel (1:1). The suspension of tumor cells was injected intraperitoneally, approximately 0.5 cm above the midpoint of the inguinal ligament.

To assess the role of tumor-derived exosomes in peritoneal metastasis, exosomes (50 μg in 200 μL PBS) isolated from conditioned media of AGS cells overexpressing FERMT2 or carrying a control vector were injected into the peritoneal cavity of BALB/c nude mice. Additionally, the TGF-β receptor kinase inhibitor SB-431542 (10 μM, diluted in 1 μL DMSO) was administered intraperitoneally on days 1, 3, 5, and 7 after tumor cell injection, in a volume of 200 μL PBS per dose. After 40 days, the mice were euthanized. Intraperitoneal tumor nodules were collected, photographed, weighed, and subjected to statistical analysis. The nodules were then fixed in paraffin and sectioned for IHC staining. All animal procedures were approved by the Ethics Committee of the First Affiliated Hospital of Zhejiang University (Approval No.2025-116).

### Statistics analysis

All experiments were performed in a minimum of three independent replicates. Data are expressed as the mean ± standard error. Statistical comparisons between two groups were carried out using the two-tailed Student's t-test. One-way ANOVA followed by Bonferroni's post-hoc test was applied to assess differences among three or more groups. A P-value less than 0.05 was considered statistically significant ((^ns^p>0.05; *p < 0.05; **p < 0.01; ***p < 0.001; ****p < 0.0001).

## Results

### *FERMT2* expression is positively associated with CAF infiltration and maintains myofibroblastic phenotype in GCAFs

**Figure 1:**
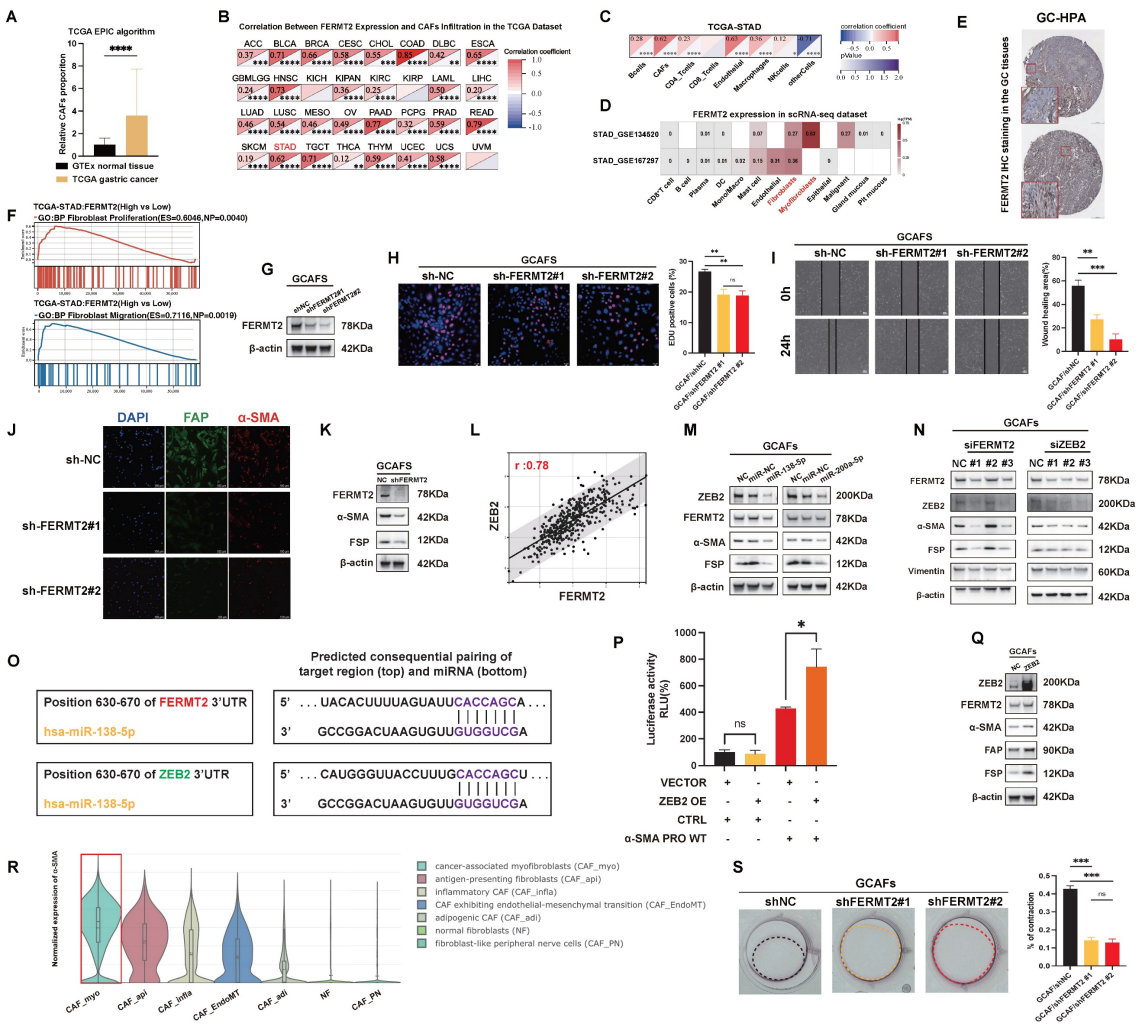
***FERMT2* expression correlates with CAF infiltration and sustains the myofibroblastic phenotype of GCAFs.** (A) EPIC algorithm analysis of TCGA-STAD and GTEx datasets showing a significantly higher proportion of CAFs in gastric cancer tissues compared with normal tissues. (B) Correlation heatmap depicting positive associations between *FERMT2* expression and CAF infiltration across multiple TCGA cancer types. (C) In TCGA-STAD, *FERMT2* expression exhibits the second strongest correlation with CAFs infiltration among all analyzed cell types. (D) Single-cell RNA-seq datasets (GSE154352, GSE167297) reveal predominant *FERMT2* expression in fibroblast clusters within gastric cancer tissues. (E) Representative IHC images from the HPA database showing *FERMT2* expression in gastric cancer tissues. (F) GSEA demonstrating significant enrichment of fibroblast proliferation and migration gene signatures in tumors with high *FERMT2* expression (TCGA-STAD cohort). (G) Immunoblot analysis of FERMT2 in GCAFs with stable *FERMT2* knockdown. (H) EdU incorporation assay showing reduced proliferation in *FERMT2*-depleted GCAFs. (I) Wound-healing assay demonstrating impaired migration following *FERMT2* knockdown. (J) Immunofluorescence staining showing decreased α-SMA (red) and FAP (green) levels upon *FERMT2* knockdown; nuclei counterstained with DAPI. (K) Western blot analysis showing reduced *α-SMA* and *FSP* expression in *FERMT2*-depleted GCAFs. (L) Pearson correlation analysis in TCGA-STAD showing a strong positive association between *FERMT2* and *ZEB2* (r = 0.78). (M) *miR-200a* and *miR-138* simultaneously target *FERMT2* and *ZEB2*, suppressing *α-SMA* and *FSP* expression. (N) Silencing either *FERMT2* or *ZEB2* reduces expression of the other, as well as *α-SMA*, *FSP*, and *vimentin* in GCAFs. (O) TargetScan-predicted *miR-138-5p* binding sites within the 3′UTRs of both *FERMT2* and *ZEB2*. (P) Dual-luciferase reporter assay showing that *ZEB2* overexpression activates *α-SMA* promoter activity. (Q) Immunoblot showing upregulated *α-SMA*, *FAP*, and *FSP* in *ZEB2*-overexpressing GCAFs. (R) Violin plot of scRNA-seq data showing enrichment of *α-SMA* expression in CAF_myo compared with other CAF subtypes and NFs. (S) Collagen contraction assay showing reduced contractility in *FERMT2*-knockdown GCAFs; contraction quantified using ImageJ. (Statistical significance: ns, p > 0.05; *p < 0.05; **p < 0.01; ***p < 0.001; ****p < 0.0001.)

To investigate the stromal characteristics of GC, we analyzed TCGA-STAD transcriptomic data using the EPIC algorithm and observed significantly higher CAF infiltration in tumor tissues compared to adjacent normal tissues (Fig. 1A). Our prior single-cell transcriptomic study across multiple cancer types identified *FERMT2* as predominantly expressed in stromal components, particularly in CAFs 15. Consistent with this, Spearman correlation analysis across TCGA pan-cancer cohorts revealed a strong positive association between *FERMT2* expression and CAF infiltration in multiple tumor types (Fig. 1B). In GC specifically, *FERMT2* expression correlated positively with CAF and endothelial cell infiltration, while associations with other immune subsets were minimal or absent (Fig. 1C).

Single-cell RNA-sequencing data from the TISCH database further confirmed that *FERMT2* expression was enriched in gastric CAFs (Fig. 1D). Supporting this, immunohistochemical data from the Human Protein Atlas (HPA) showed *FERMT2* staining was largely restricted to stromal regions in gastric cancer tissues (Fig. 1E). Collectively, these findings establish *FERMT2* as a stromal-enriched gene, closely associated with CAF infiltration, highlighting its potential as a CAF marker and functional regulator.

To explore its functional role, we stratified TCGA-STAD samples by *FERMT2* expression and observed that high *FERMT2* levels were associated with transcriptomic signatures related to fibroblast proliferation and migration (Fig. 1F). We next generated stable *FERMT2* knockdown GCAF lines and confirmed effective silencing at the protein level (Fig. 1G). Functional assays revealed that *FERMT2* silencing impaired both migratory and proliferative capacities of GCAFs (Fig. 1H-I). Immunofluorescence staining demonstrated reduced expression of CAF activation markers, including *FAP* and *α-SMA*, upon *FERMT2* depletion (Fig. 1J), which was corroborated by Western blot analysis (Fig. 1K). Together, these data implicate *FERMT2* as a key regulator of CAF activation and the acquisition of tumor-promoting traits.

Activation of CAFs refers to the transition from a quiescent to a functionally active state, marked by changes in cellular function and phenotype. A hallmark of CAF activation is the acquisition of a myofibroblastic phenotype, characterized by increased *α-SMA* expression and cytoskeletal remodeling that enhances contractility and ECM remodeling 16,17. Within the TCGA-STAD cohort, *FERMT2* expression showed a strong positive correlation with *ZEB2*, a transcription factor linked to myofibroblastic transition (Fig. 1L).

In GCAFs, transfection with *miR-138* or *miR-200a* led to downregulation of both *FERMT2* and *ZEB2*, accompanied by reduced expression of *α-SMA* and *FAP* (Fig. 1M). To assess whether *FERMT2* and *ZEB2* function in a competing endogenous RNA network, we performed reciprocal knockdowns. Silencing *FERMT2* led to reduced *ZEB2* protein levels, and vice versa (Fig. 1N). TargetScan analysis identified a shared *miR-138-5p* binding site in the 3′UTRs of both *FERMT2* and *ZEB2* (Fig. 1O), supporting the hypothesis of post-transcriptional competition.

Using dual-luciferase reporter assays, we confirmed that *ZEB2* binds to the *ACTA2* (*α-SMA*) promoter and transcriptionally activates its expression (Fig. 1P). *ZEB2* overexpression in GCAFs enhanced *α-SMA* protein levels and upregulated additional CAF activation markers, including *FAP* and *FSP* (Fig. 1Q). Single-cell RNA-sequencing (scRNA-seq) data from the Cancer-Associated Fibroblast Atlas revealed that *α-SMA* expression is predominantly enriched in the myofibroblastic CAF subset (CAF_myo) (Fig. 1R), consistent with the phenotype observed in our study. Notably, contractile activity was significantly impaired in *FERMT2*-depleted GCAFs (Fig. 1S).

Taken together, these findings indicate that *FERMT2* sustains the myofibroblastic phenotype of GCAFs by functioning as a ceRNA for *ZEB2*. Through *ZEB2*-mediated transcriptional activation of *α-SMA*, *FERMT2* facilitates the activation, migration, and contractility of GCAFs, thereby contributing to tumor-stroma crosstalk and a pro-tumorigenic microenvironment.

### *FERMT2*⁺ CAFs display distinct transcriptional states and enhanced crosstalk with tumor cells via *TGF-β* signaling

**Figure 2:**
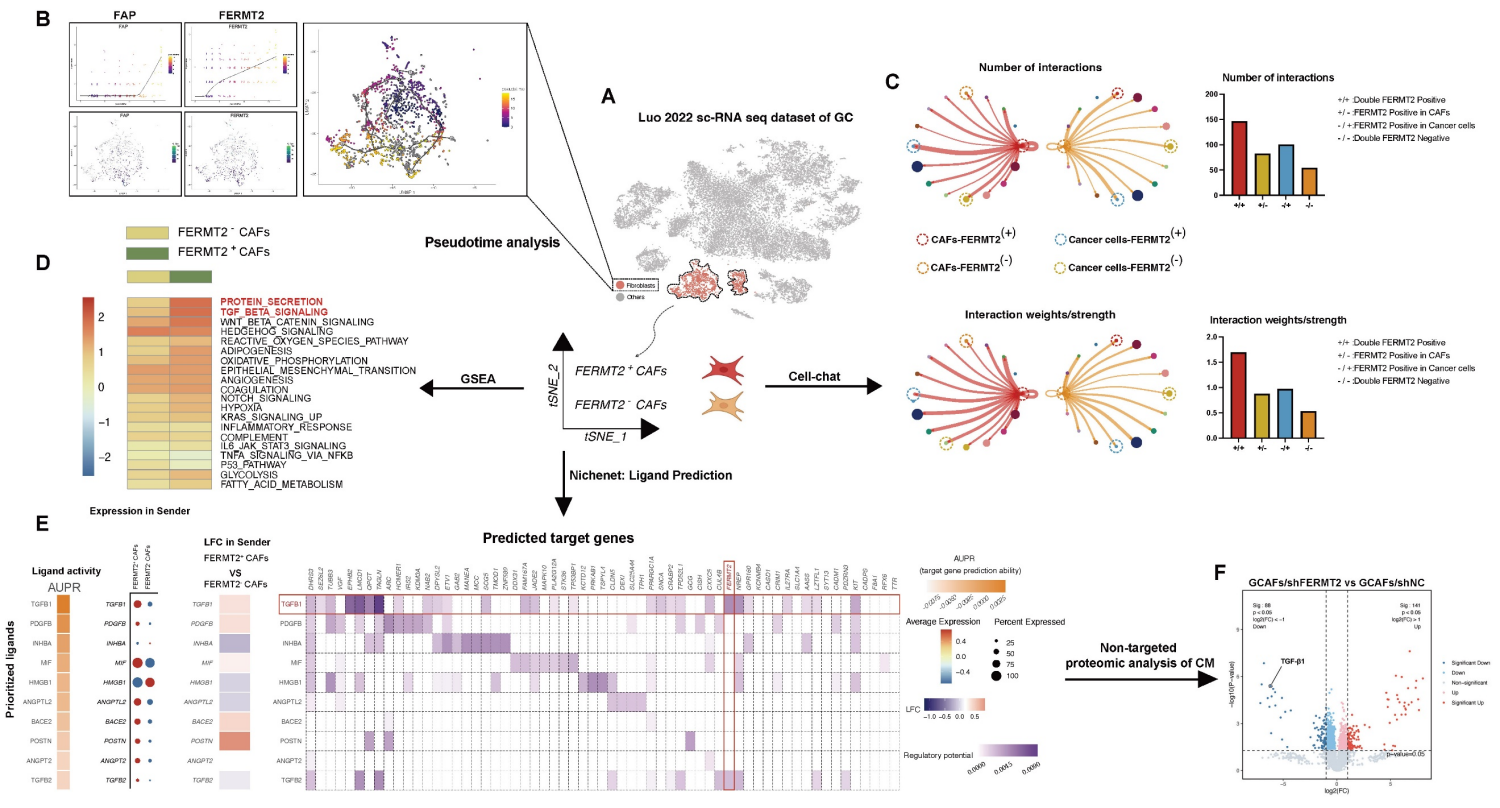
***FERMT2*⁺ CAFs exhibit distinct transcriptional programs and enhanced communication with cancer cells via *TGF-β* signaling.** (A) Schematic overview of the analysis pipeline using the gastric cancer single-cell RNA-seq dataset (Luo et al., 2022). Fibroblast clusters were isolated for pseudotime, CellChat, GSEA, and NicheNet analyses based on *FERMT2* expression. (B) Pseudotime analysis of CAF populations showing dynamic expression patterns of *FAP* and *FERMT2* along the trajectory. UMAP plot illustrates clustering and annotation of CAFs in different states. (C) Cell-cell communication analysis using CellChat reveals that *FERMT2*⁺ CAFs exhibit more interactions and stronger signaling intensity with cancer cells than *FERMT2*⁻ CAFs. Right panels: Quantification of interaction numbers and signaling strength between *FERMT2*⁺/⁻ CAFs and cancer cells, with or without *FERMT2* expression. (D) GSEA comparing *FERMT2*⁺ versus *FERMT2*⁻ CAFs reveals enrichment of pro-tumorigenic pathways, including TGF-β, WNT, and EMT signaling. (E) NicheNet analysis identifies key ligands involved in CAF-cancer cell communication. Heatmaps and dot plots display ligand activity (AUPR), expression changes in CAFs, and predicted regulatory potential of target genes. Notably, TGF-β1 is significantly upregulated in *FERMT2*⁺ CAFs. (F) Volcano plot of untargeted proteomic analysis of CM from GCAFs after *FERMT2* knockdown versus control (sh-NC). Downregulation of secreted TGF-β1 and other associated proteins indicates *FERMT2*-dependent regulation of secretion.

We interrogated the gastric cancer scRNA-seq dataset (Luo, 2022) to characterize GCAF heterogeneity based on *FERMT2* expression. GCAFs were stratified into *FERMT2*⁺ and *FERMT2*⁻ subpopulations (Fig. 2A). Pseudotime trajectory analysis revealed a progressive upregulation of both *FERMT2* and *FAP* along the differentiation axis, suggesting a role for *FERMT2* in driving GCAF activation or phenotypic transitions (Fig. 2B). Notably, the *FERMT2* double-negative group (*FERMT2*⁻ GCAFs / *FERMT2*⁻ GC cells) exhibited the fewest and weakest cell-cell interactions (Fig. 2C). In addition, *FERMT2*⁻ GCAFs showed markedly diminished secretory capacity and reduced *TGF-β* pathway activity (Fig. 2D), implying impaired secretion of key factors such as TGF-β1 and attenuated support for tumor progression.

NicheNet analysis identified TGF-β1 as the most significantly altered ligand between *FERMT2*⁺ and *FERMT2*⁻ GCAFs (Fig. 2E), a finding validated by untargeted proteomics of conditioned media from GCAFs with or without *FERMT2* knockdown (Fig. 2F). Collectively, these results suggest that *FERMT2* promotes GCAF activation and augments communication with gastric cancer cells, likely through enhanced secretion of pro-tumorigenic mediators such as TGF-β1.

### *FERMT2*^+^ GCAFs enhance gastric cancer cell migration, invasion, and anoikis resistance via TGF-β1 secretion

**Figure 3:**
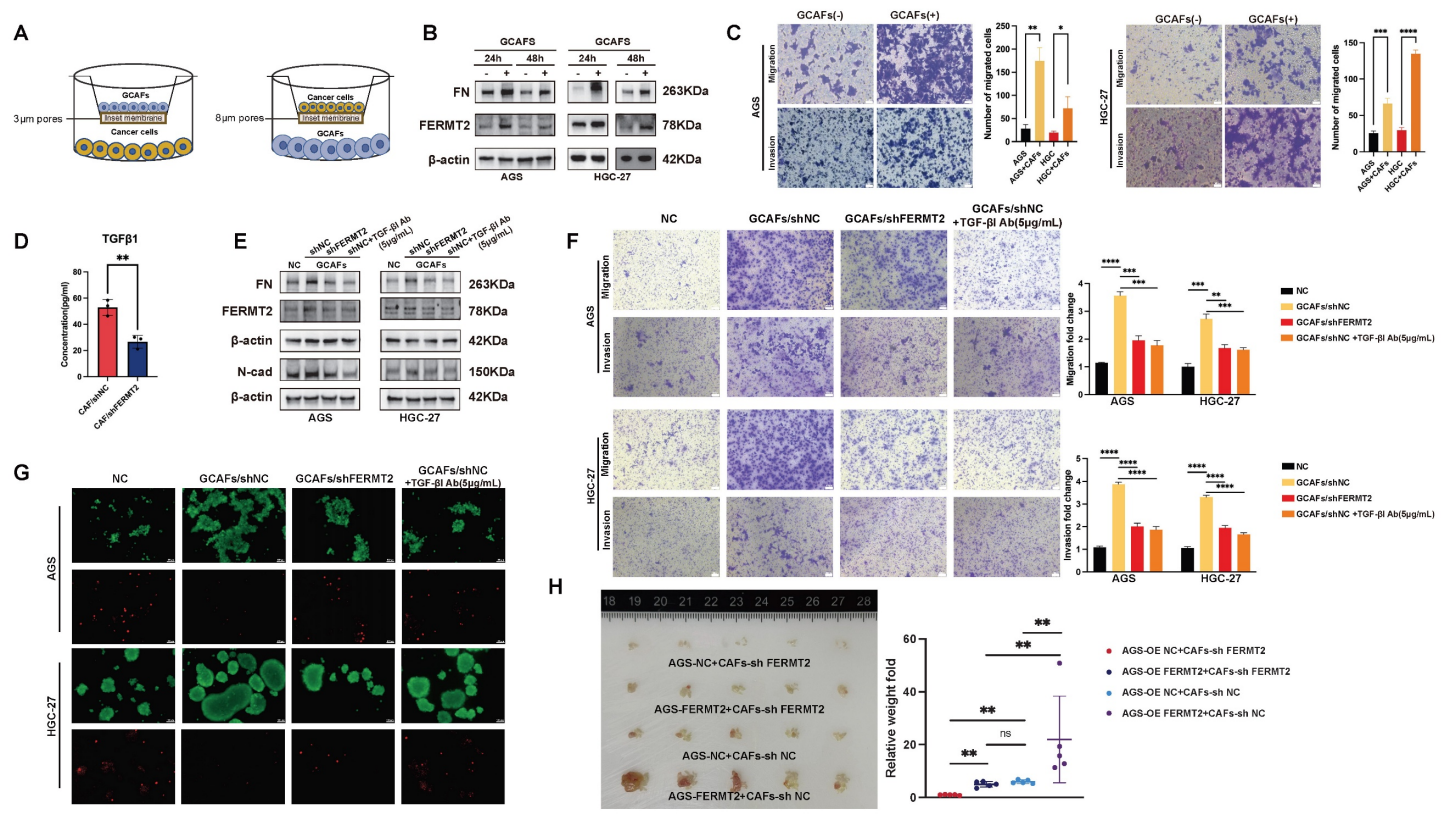
***FERMT2*-expressing CAFs enhance gastric cancer cell migration, invasion, and anoikis resistance via TGF-β1 secretion.** (A) Schematic illustration of the transwell co-culture system used to assess the impact of GCAFs on protein expression in GC cells (3 μm pores) and on cancer cell migration and invasion (8 μm pores). (B) Western blot analysis of *FN* and *FERMT2* expression in AGS and HGC-27 cells co-cultured with or without GCAFs for 24 and 48 hours. (C) Migration and invasion assays showing significant promotion of AGS and HGC-27 cell motility by GCAFs. (D) ELISA analysis of TGF-β1 levels in conditioned media from control and *FERMT2*-knockdown GCAFs. (E) Western blot showing *FN*, *FERMT2*, and *N-cadherin* expression in AGS and HGC-27 cells cultured with sh-NC or sh-*FERMT2* GCAFs, with or without TGF-β1 neutralizing antibody (5 μg/mL). (F) Transwell migration and invasion assays demonstrating reduced GCAF-induced motility following *FERMT2* knockdown or TGF-β1 blockade. (G) Anoikis resistance assay showing that AGS and HGC-27 spheroids co-cultured with sh-NC or sh-*FERMT2* GCAFs, with or without TGF-β1 antibody, exhibit differential survival under super-low attachment conditions, evaluated by Calcein/PI staining. (H) In vivo tumorigenicity assay showing representative images and quantification of intraperitoneal tumors formed by AGS cells (OE-NC or OE-*FERMT2*) co-injected with sh-NC or sh-*FERMT2* GCAFs into nude mice. (Statistical significance: ns, p > 0.05; *p < 0.05; **p < 0.01; ***p < 0.001; ****p < 0.0001.)

Our previous work demonstrated that gastric cancer cells detached from the extracellular matrix activate autocrine *TGF-β1* signaling, leading to upregulation of *FERMT2*, increased *fibronectin* (*FN*) expression, and enhanced cell-cell adhesion to facilitate extracellular matrix deposition 18. Beyond this autocrine axis, TGF-β1 is also abundantly secreted by CAFs within the tumor microenvironment, acting in a paracrine fashion 19. To investigate this further, we established a co-culture system comprising gastric cancer cells and GCAFs. This included two experimental models: one to evaluate the impact of GCAFs on gastric cancer cell protein expression, and another to assess their effects on malignant phenotypes such as migration, invasion (Fig. 3A).

As predicted by NicheNet analysis, TGF-β1 emerged as the top-ranked differential ligand between *FERMT2*⁺ and *FERMT2*⁻ GCAFs, with *FERMT2* among its downstream targets (Fig. 2E, red rectangle). In line with this prediction, co-culture of gastric cancer cells with GCAFs markedly increased *FERMT2* and *FN* protein levels (Fig. 3B), and significantly promoted migration and invasion in both AGS and HGC-27 cell lines (Fig. 3C). ELISA confirmed that *FERMT2* knockdown in GCAFs significantly reduced TGF-β1 secretion into the extracellular milieu (Fig. 3D). Notably, co-culture with shNC-GCAFs, as opposed to sh*FERMT2*-GCAFs, robustly upregulated *FERMT2* and *FN* in gastric cancer cells and enhanced their migratory and invasive potential—effects that were abolished by a TGF-β1 neutralizing antibody (Fig. 3E, F).

Given the pivotal roles of *FERMT2* and *FN* in promoting anoikis resistance in gastric cancer 18, we next examined whether GCAF-derived TGF-β1 contribute to anchorage-independent survival. Gastric cancer cells co-cultured with control GCAFs (shNC-GCAFs) showed markedly enhanced survival under matrix detachment compared with those co-cultured with *FERMT2*-deficient GCAFs (Fig. 3G). Consistently, in vivo, these cells developed more extensive peritoneal dissemination in BALB/c nude mice (Fig. 3H). Together, these findings demonstrate that *FERMT2* promotes GCAF-mediated paracrine secretion of TGF-β1, which in turn drives the invasive behavior and anoikis resistance of gastric cancer cells.

### *FERMT2* regulates exosomal transfer of COL6A1 to enhance *TGF-β* signaling and CAF activation

**Figure 4:**
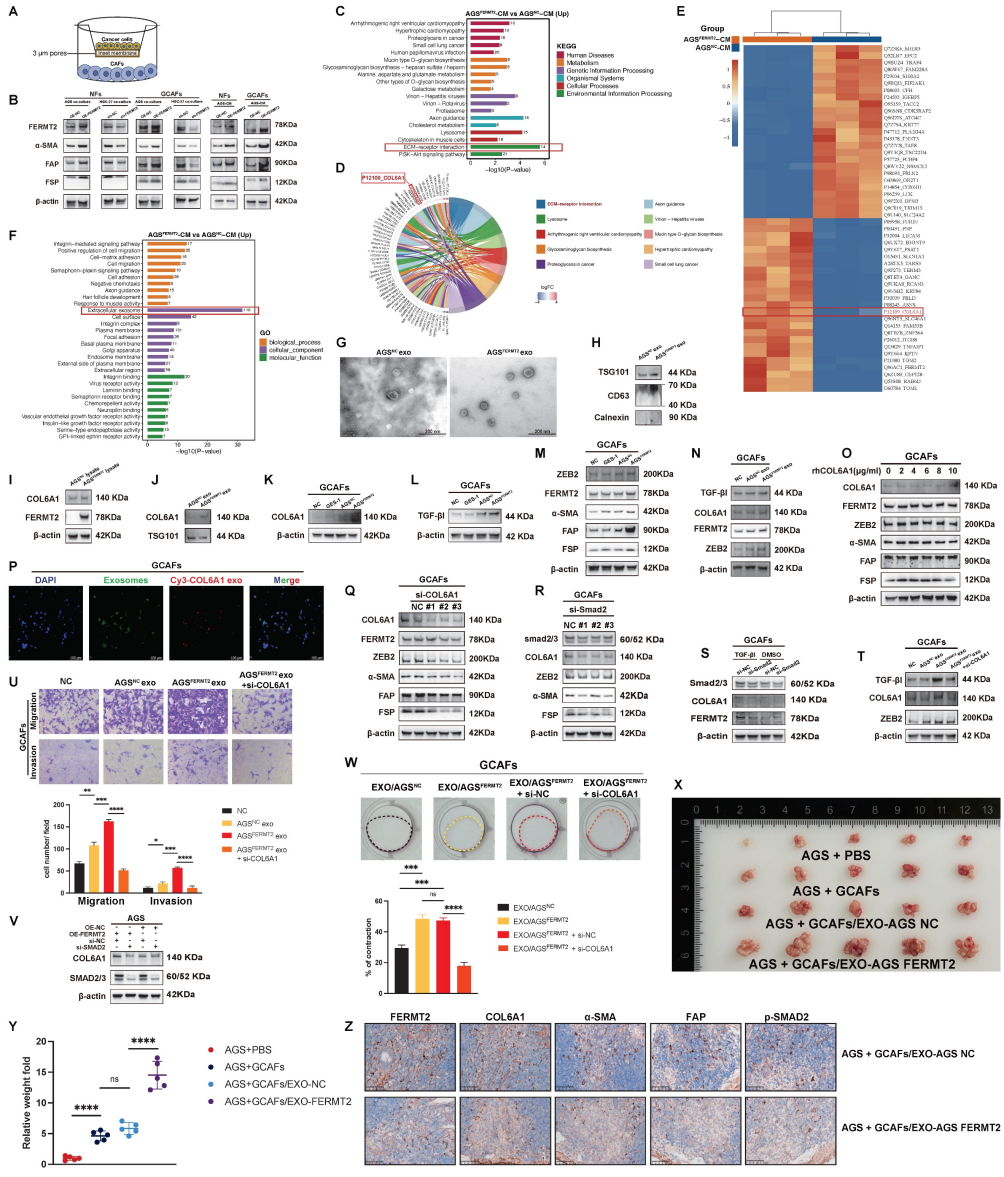
***FERMT2* regulates exosomal transfer of COL6A1, enhancing *TGF-β* signaling and CAF activation.** (A) Schematic illustration of the transwell co-culture system (3 μm pores) used to assess the impact of GC cells on protein expression in GCAFs. (B) Immunoblot analysis of *FERMT2*, *α-SMA*, *FAP*, and *FSP* in NFs and GCAFs co-cultured with AGS-NC, AGS-*FERMT2*, HGC-shNC, HGC-sh*FERMT2*, or with CM from AGS-NC or AGS-*FERMT2* cells. (C) KEGG pathway enrichment analysis of differentially expressed proteins in AGS-*FERMT2* CM versus AGS-NC CM. (D) Functional pathway distribution of differentially expressed proteins, highlighting COL6A1 involvement in ECM-receptor interactions. (E) Top 25 up- and downregulated proteins in AGS-*FERMT2* CM versus AGS-NC CM. (F) GO analysis showing the most enriched pathways in AGS-*FERMT2* CM versus AGS-NC CM. (G) Transmission electron microscopy (TEM) images of exosomes from AGS-NC and AGS-*FERMT2* cells. (H) Immunoblot analysis of exosomal markers (*TSG101*, *CD63*) and the endoplasmic reticulum marker (*Calnexin*) in exosomes from AGS-NC and AGS-*FERMT2* cells. (I) *COL6A1* expression in lysates of AGS-NC and AGS-*FERMT2* cells. (J) Immunoblot showing *COL6A1* and *TSG101* expression in exosomes from AGS-NC and AGS-*FERMT2* cells. (K-M) Western blot analysis of *COL6A1* (K), *TGF-β1* (L), and *ZEB2*, *FERMT2*, *α-SMA*, *FAP*, and *FSP* (M) in GCAFs co-cultured with GES-1, AGS-NC, or AGS-*FERMT2* cells. (N) Immunoblot analysis of *TGF-β1*, *COL6A1*, *FERMT2*, and *ZEB2* in GCAFs treated with exosomes from AGS-NC or AGS-*FERMT2* cells. (O) Western blot showing *COL6A1*, *FERMT2*, *ZEB2*, *α-SMA*, *FAP*, and *FSP* in GCAFs treated with rhCOL6A1 at different concentrations for 24 h. (P) Co-localization of exosomes and COL6A1 in GCAFs after 24 h incubation with double-labeled exosomes (PKH67, green; COL6A1, Cy3, red; nuclei, DAPI, blue). Scale bar, 100 μm. (Q) Western blot showing *COL6A1*, *FERMT2*, *ZEB2*, *α-SMA*, *FAP*, and *FSP* expression in GCAFs after *COL6A1* knockdown. (R, S) Western blot analysis of *Smad2/3*, *COL6A1*, *ZEB2*, *α-SMA*, and *FSP* after siRNA-mediated silencing of *Smad2/3* (R) and assessment of *Smad2/3*-mediated *TGF-β1* signaling on *COL6A1* and *FERMT2* expression (S) in GCAFs. (T) Western blot showing *TGF-β1*, *COL6A1*, and *ZEB2* in GCAFs treated with exosomes from AGS-NC or AGS-*FERMT2* cells, with or without si-*COL6A1*. (U) Transwell migration and invasion assays of GCAFs treated with exosomes from AGS-NC or AGS-*FERMT2* cells, with or without *COL6A1* knockdown. (V) *COL6A1* and *Smad2/3* expression in AGS cells with *FERMT2* overexpression or knockdown of *Smad2*. (W) Collagen gel contraction assay of GCAFs treated with exosomes from indicated GC cells, with or without *COL6A1* knockdown; contraction quantified using ImageJ. (X, Y) In vivo tumorigenicity assays showing representative images and quantification of intraperitoneal tumors formed by AGS cells co-injected with GCAFs, with or without tumor-derived exosomes, into nude mice. (Z) Immunohistochemical staining of peritoneal metastatic nodules from BALB/c nude mice. (Statistical significance: ns, p > 0.05; *p < 0.05; **p < 0.01; ***p < 0.001; ****p < 0.0001.)

In preceding sections, we showed that *FERMT2* promotes GCAF activation and facilitates their interaction with gastric cancer cells. However, intercellular communication within the tumor microenvironment is bidirectional. In breast cancer, for example, tumor-derived *FERMT2* promotes TAM infiltration by upregulating CSF1 secretion 10. To investigate whether a similar mechanism exists in gastric cancer, we established a co-culture model to examine the influence of tumor cells on GCAFs (Fig. 4A). Both co-culture and treatment with GC cell-derived conditioned media (CM) showed that AGS cells overexpressing *FERMT2* markedly upregulated *FERMT2* and CAF activation markers in GCAFs and NFs, whereas *FERMT2* knockdown in HGC-27 cells attenuated these effects (Fig. 4B).

*FERMT2* is a critical mediator of integrin activation, known to regulate cell adhesion and focal adhesion signaling 20. Proteomic profiling of CM from gastric cancer cells with differential *FERMT2* expression revealed significant enrichment of the ECM-receptor interaction pathway in the *FERMT2*-overexpressing group (Fig. 4C). Among the top 20 differentially secreted proteins, COL6A1 was prominently associated with this pathway (Fig. 4D, E). Moreover, gene ontology analysis indicated a striking enrichment of extracellular exosome-related components in the *FERMT2*-overexpressing CM, suggesting a role for exosome-mediated communication (Fig. 4F).

Transmission electron microscopy confirmed the characteristic morphology of exosomes isolated from CM (Fig. 4G), and expression of canonical markers *TSG101* and *CD63* validated their identity (Fig. 4H). *FERMT2* overexpression significantly increased *COL6A1* protein levels in AGS cells (Fig. 4I) and enhanced its loading into exosomes (Fig. 4J). Co-culture with *FERMT2*-overexpressing AGS cells also elevated *COL6A1* expression in GCAFs compared to controls or normal gastric epithelial cells (Fig. 4K).

*COL6A1* has been implicated in potentiating TGF-β signaling and tumor metastasis 21. In line with this, co-culture with *FERMT2*-overexpressing AGS cells markedly increased the expression of *TGF-β1*, *ZEB2*, *FERMT2*, and CAF activation markers in GCAFs (Fig. 4L, M). Similar effects were induced by exosomes from *FERMT2*-overexpressing cells, which also elevated *COL6A1* and *TGF-β1* levels in GCAFs (Fig. 4N).

To determine whether COL6A1 acts extracellularly or intracellularly, we supplemented GCAF cultures with recombinant COL6A1 protein. This failed to induce CAF activation or internalization (Fig. 4O). In contrast, PKH67-labeled exosomes transferred Cy3-labeled COL6A1 into GCAFs (Fig. 4P), supporting an intracellular delivery mechanism via exosomes. Thus, COL6A1 may be transferred from GC cells to CAFs via exosomes, serving as key mediators of genetic communication between cancer cells and the stroma.

Knockdown of *COL6A1* in GCAFs reduced the expression of *ZEB2*, *FERMT2*, and CAF activation markers (Fig. 4Q). Silencing *SMAD2*—a core *TGF-β* signaling effector—similarly decreased *ZEB2* and CAF marker expression (Fig. 4R). Interestingly, *SMAD2* knockdown also lowered *COL6A1* levels, suggesting a positive feedback loop in which COL6A1 enhances TGF-β autocrine signaling to sustain its own expression (Fig. 4S). Moreover, *COL6A1* depletion partially reversed the upregulation of *TGF-β1* in GCAFs (Fig. 4T), of which the exosome-induced migration and invasion were attenuated in the absence of *COL6A1* (Fig. 4U).

*FERMT2* (also known as *Kindlin-2*) has been reported to regulate both actin-binding capacity and the activity of TGF-β receptor 1 (*TGFβR1*) 22. In our study, *SMAD2* knockdown abolished the *FERMT2*-induced upregulation of *COL6A1* (Fig. 4V), indicating that this regulation is dependent on enhanced *TGF-β* signaling. Exosomes derived from *FERMT2*-overexpressing gastric cancer cells increased the contractile activity of GCAFs, whereas *COL6A1* silencing in GCAFs markedly attenuated this effect (Fig. 4W). In vivo, tumors formed following injection of AGS-*FERMT2*-derived exosomes displayed a stronger peritoneal metastatic capacity, along with markedly higher staining intensities of FERMT2, COL6A1, phosphorylated SMAD2/3, and CAF activation markers (α-SMA and FAP), compared with those formed with AGS-NC-derived exosomes (Fig. 4X-Z). Collectively, these findings demonstrate that gastric cancer cells deliver COL6A1 to GCAFs via exosomes, thereby amplifying *TGF-β* signaling and sustaining CAF activation.

### Characterization of *COL6A1* and *ZEB2* expression in gastric cancer and their association with peritoneal metastasis and survival

**Figure 5:**
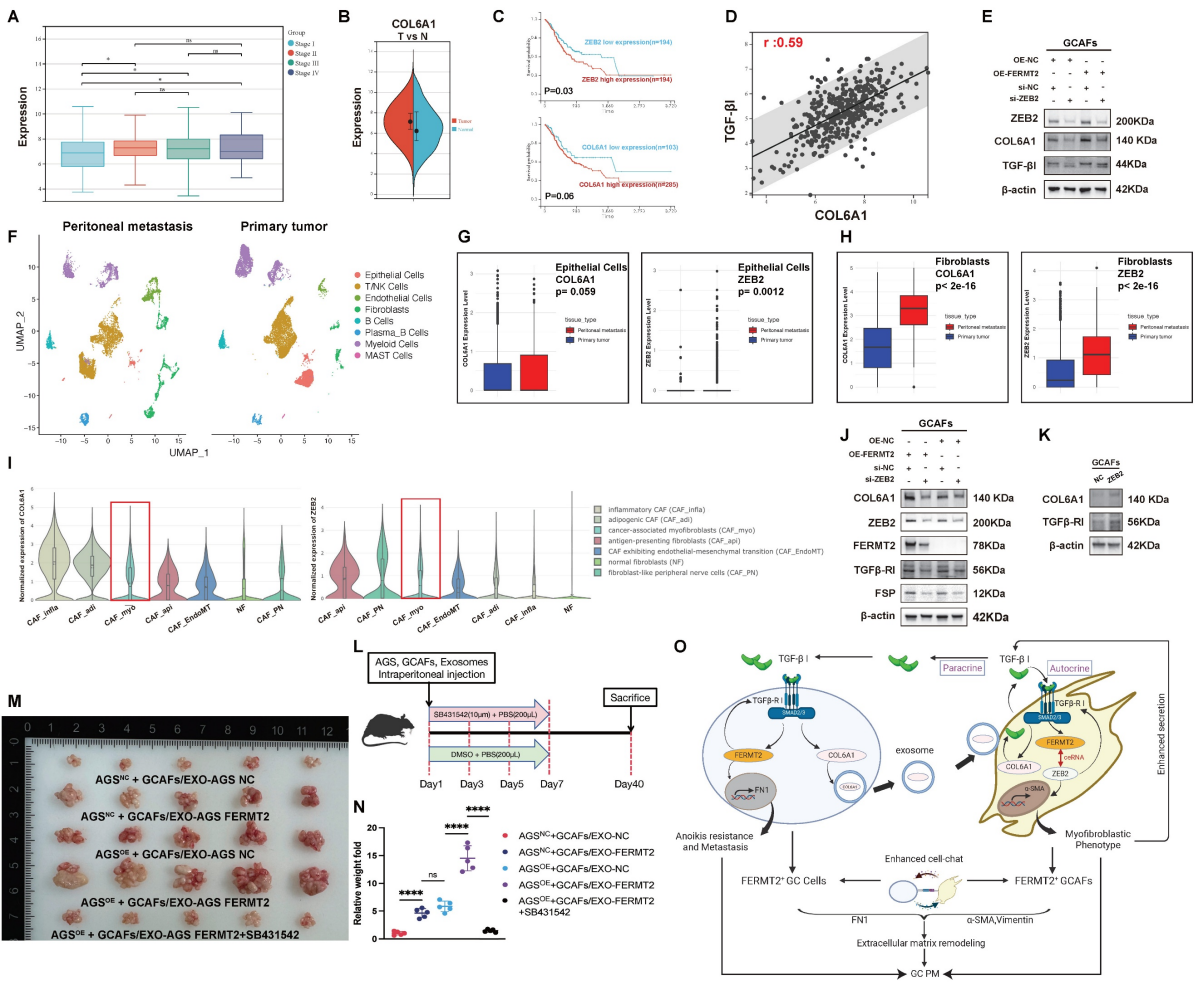
** Characterization of *COL6A1* and *ZEB2* expression in gastric cancer and their association with peritoneal metastasis and survival.** (A) Expression of *COL6A1* across different stages of gastric cancer. (B) *COL6A1* expression in gastric cancer tissues compared to normal adjacent tissues. (C) Kaplan-Meier survival analysis comparing overall survival in gastric cancer patients with high versus low expression of *ZEB2* and *COL6A1*. (D) Correlation analysis between *COL6A1* and *TGF-β1* expression in gastric cancer tissues. (E) Expression of *ZEB2*, *COL6A1*, and *TGF-β1* in GCAFs following *FERMT2* overexpression or *ZEB2* knockdown. (F) UMAP plot showing cell type clustering in gastric cancer tissues, including both peritoneal metastasis and primary tumor tissues. (G) Comparison of *COL6A1* and *ZEB2* expression in epithelial cells from primary tumors and peritoneal metastasis tissues. (H) Comparison of *COL6A1* and *ZEB2* in fibroblasts from primary tumors and peritoneal metastasis tissues. (I) Violin plots illustrating the normalized expression of *COL6A1* and *ZEB2* across different CAF subtypes. (J) Expression of *COL6A1*, *ZEB2*, *TGFβ-RI*, and *FSP* in GCAFs treated with either *FERMT2* overexpression or control, and *ZEB2* knockdown or control. (K) Western blot analysis showing *COL6A1* and *TGFβ-RI* expression in GCAFs after *ZEB2* overexpression or control treatment. (L) Schematic showing the administration of TGF-β receptor kinase inhibitor SB-431542 into the peritoneal cavity of nude mice after tumor cell injection. (M, N) Representative images and quantification of intraperitoneally injected tumors formed by indicated cell mixtures and tumor-derived exosomes, with or without TGF-β receptor kinase inhibitor, in nude mice. (O) Schematic model summarizing the findings of this study. (Statistical significance: ns, p > 0.05; *p < 0.05; **p < 0.01; ***p < 0.001; ****p < 0.0001.)

We first examined the expression of *COL6A1* across different stages of GC. *COL6A1* expression was significantly higher in advanced stages compared to earlier stages (Fig. 5A). Moreover, *COL6A1* levels were elevated in tumor tissues relative to adjacent normal tissues (Fig. 5B). Survival analysis revealed that high *ZEB2* expression was associated with poorer survival outcomes. Although *COL6A1* expression did not reach statistical significance in survival analysis, there was a trend towards higher expression in patients with worse outcomes (Fig. 5C). Correlation analysis further indicated a positive association between *COL6A1* and *TGF-β1* expression (r = 0.59), suggesting a potential link between *COL6A1* and *TGF-β* signaling in GC (Fig. 5D).

We next explored the molecular regulation of *COL6A1* and *ZEB2* expression. Overexpression of *FERMT2* led to an increase in *ZEB2*, *COL6A1*, and *TGF-β1* protein levels. Silencing *ZEB2* abolished the *FERMT2*-induced upregulation of *COL6A1* and *TGF-β1*, indicating that *ZEB2* is essential for the *FERMT2*-mediated regulation of these proteins (Fig. 5E).

scRNA-seq of GC samples revealed distinct cell clusters within the TME, with clear separation between primary tumor and peritoneal metastasis tissues (Fig. 5F). Further analysis of *COL6A1* and *ZEB2* expression in various cell populations showed that both *COL6A1* and *ZEB2* were more highly expressed in epithelial cells and fibroblasts from peritoneal metastases compared to primary tumors (Fig. 5G, H).

Additionally, the Single Cell Cancer-Associated Fibroblasts Atlas highlighted that both *COL6A1* and *ZEB2* were enriched in the CAF_myo subset (Fig. 5I). In GCAFs, overexpression of *FERMT2* significantly upregulated *COL6A1* expression, which was partially reversed by *ZEB2* knockdown. This suggests that *FERMT2* regulates *COL6A1* expression via a *ZEB2*-dependent mechanism. A similar trend was observed for *TGFβ-RI* expression (Fig. 5J). Moreover, *ZEB2* overexpression in GCAFs increased the protein levels of both *COL6A1* and *TGFβ-RI*, indicating that *ZEB2*-induced *COL6A1* upregulation is mediated by the upregulation of *TGFβ-RI* (Fig. 5K).

To assess the role of TGF-β receptor kinase inhibitor SB-431542 in peritoneal metastasis, which was administered intraperitoneally on days 1, 3, 5, and 7 after tumor cell injection (Fig. 5L). And we found the treatment with SB431542 inhibited peritoneal tumor formation ability in AGS cells together with GCAFs (Fig. 5M, N). Finally, the proposed model of this study is illustrated in Fig. 5O.

## Discussion

The role of *FERMT2* in CAF activation has remained incompletely defined. MicroRNAs (miRNAs), a class of small non-coding RNAs, modulate gene expression by binding to the 3′ untranslated region (3′-UTR) of target mRNAs, thereby influencing multiple stages of tumorigenesis.

*MiR-138-5p* has been reported to suppress cancer cell functions through diverse mechanisms, ultimately impeding tumor progression 23,24. For instance, *miR-138-5p* delivered via mesenchymal stem cell (MSC)-derived exosomes targets *SIRT1*, attenuating dermal fibroblast proliferation and migration while reducing *NF-κB*, *α-SMA*, and *TGF-β1* expression 25. Likewise, the *miR-200* family shapes the tumor microenvironment by targeting *ZEB2* to inhibit epithelial-mesenchymal transition (EMT) 26,27, and *miR-200b* directly suppresses *FERMT2*, thereby impacting cytoskeletal remodeling and adhesion 28. Notably, in oral squamous cell carcinoma, *FERMT2* upregulates *ZEB2* to promote tumor cell migration and invasion 29.

In parallel, studies in pulmonary fibrosis have shown that *ZEB2* deletion reduces *α-SMA* and *vimentin* expression, thereby reversing the fibrotic phenotype 30. In the tumor microenvironment, CAFs—major stromal constituents—express markers including *FAP*, *vimentin*, *S100A4*, and *α-SMA*, and secrete matrix metalloproteinases such as MMP9 to remodel the extracellular matrix 31. Among subsets of CAFs, myofibroblastic CAFs (CAF_myo) display elevated *α-SMA* levels, heightened contractile capacity, and a stronger propensity for ECM remodeling 32.

Our findings identify a previously uncharacterized regulatory circuit in which *FERMT2* functions as a ceRNA for *ZEB2* in GCAFs, counteracting repression by *miR-200a* and *miR-138*. This axis enhances *ZEB2* expression, leading to *α-SMA* upregulation and augmented contractility, thereby sustaining the myofibroblastic CAF phenotype. Such phenotypic reprogramming facilitates the transition of CAFs from a quiescent to an active state and plays a critical role in fostering peritoneal metastasis in gastric cancer.

Once functionally activated, CAFs secrete a wide range of signaling molecules, including cytokines, chemokines, proteins, and exosomes, collectively forming a distinct "secretome" that actively shapes the tumor microenvironment 33,34. Among these factors, TGF-β1 is produced not only by tumor cells but also by stromal components such as CAFs 35,36. TGF-β1 plays a pivotal role in sustaining cancer stemness 37-39, while also driving EMT processes in cancer cells 40,41, which enhances resistance to anoikis and therapeutic interventions and contributes to metastatic progression.

This secretome mediates intricate intercellular communication among CAFs, cancer cells, immune cells, and endothelial cells through both autocrine and paracrine signaling pathways 19. In this study, we found that TGF-β1 secreted by CAFs was taken up by gastric cancer cells via TGFβ-RI, leading to the activation of the downstream *SMAD2/3*-*FERMT2* signaling cascade. Notably, the upregulation of *FERMT2*, in turn, enhanced the expression of *TGFβ-RI*, thereby further amplifying *TGF-β1* signaling and establishing a positive feedback loop. This TGF-β1-mediated paracrine signaling also promoted the expression of *FN* in gastric cancer cells, which enhanced cell-cell adhesion and ECM deposition, ultimately contributing to increased resistance to anoikis.

However, intercellular communication within the tumor immune microenvironment is not unidirectional 42; rather, cancer cells can reciprocally modulate the functional state of CAFs through various mechanisms, including the secretion of exosomes. Enhanced *TGF-β1* signaling in cancer cells promotes the expression of *COL6A1* and increases exosome release, facilitating the transfer of COL6A1-enriched exosomes to GCAFs. Upon uptake, GCAFs exhibiting elevated *COL6A1* levels further intensify their TGF-β1 secretion, as previously reported 21, leading to the acquisition of a myofibroblastic phenotype characterized by upregulated *α-SMA* and *vimentin* expression via TGF-β1 autocrine signaling. This phenotypic transition promotes ECM remodeling, thereby contributing to the establishment of a pro-metastatic microenvironment. Moreover, activated CAFs exhibit upregulated expression of *FERMT2* and *ZEB2*, and secrete increased levels of TGF-β1, which in turn enhances the anoikis resistance and migratory potential of gastric cancer cells. Collectively, this reciprocal positive feedback loop amplifies *TGF-β1* signaling between CAFs and cancer cells, ultimately promoting peritoneal metastasis in gastric cancer.

In this study, we demonstrate that exosomes efficiently transport COL6A1 to GCAFs. This finding supports the role of exosomes as effective carriers of intercellular communication, capable of traversing the cell membrane and delivering cargo such as COL6A1 to specific subcellular compartments 43. In contrast, recombinant COL6A1 failed to exhibit similar internalization in GCAFs, suggesting that its ineffective uptake may be due to structural differences compared to exosome-derived COL6A1. During the packaging and secretion of exosomes, proteins may undergo specific modifications, such as glycosylation or phosphorylation, which can enhance their interaction with cell surface receptors, thereby facilitating endocytosis 44. Exosomal COL6A1 is likely recognized and internalized through receptor-mediated endocytosis, directing it to particular intracellular compartments. However, recombinant COL6A1 may lack these modifications, leading to its inefficient internalization. Furthermore, exosome uptake typically occurs via clathrin-mediated or caveolae-dependent endocytosis, both of which are receptor-specific processes 45. The failure of recombinant COL6A1 to interact with these receptors likely explains its inability to effectively enter the cells.

Previous studies have shown that CAF-derived IL-33 promotes peritoneal dissemination by activating the *ST2L*-*ERK1/2*-*SP1*-*ZEB2* axis, thereby inducing EMT in gastric cancer cells 46. Moreover, immune cells within ascitic fluid can drive TAMs to shift from a *CTS*^high^ to a *C1Q*^high^ phenotype, leading to increased *PD-L1* and *NECTIN2* expression and facilitating immune evasion through the C1q-complement pathway 47. These observations underscore that disrupting intercellular communication within the gastric cancer immune microenvironment may represent a promising strategy to limit peritoneal metastasis.

In this context, our study identifies *FERMT2* as a pivotal regulator, orchestrating both the maintenance of the myofibroblastic phenotype in CAFs and the enhancement of anoikis resistance in gastric cancer cells. Collectively, these findings suggest that therapeutic targeting of *FERMT2* could disrupt the pro-metastatic CAF-cancer cell axis, thereby suppressing peritoneal dissemination and potentially improving clinical outcomes in gastric cancer.

Meanwhile, given the central role of the *FERMT2*-*TGF-β1*-*COL6A1* axis in promoting tumor-stroma interaction and peritoneal dissemination, targeting this pathway may offer novel therapeutic opportunities. Neutralization of TGF-β1 with monoclonal antibodies or receptor kinase inhibitors has shown promise in preclinical models to suppress CAF activation and tumor progression 48,49. In addition, inhibiting tumor-derived exosome biogenesis or uptake may disrupt the intercellular communication that reinforces this feedback loop 50. Strategies aimed at reprogramming or depleting CAFs, such as *FAP*-targeted therapies or vitamin D analogs, also hold potential for modulating the tumor microenvironment and impairing metastatic spread 51. These approaches warrant further investigation to determine their efficacy in the context of gastric cancer.

## Conclusion

In gastric cancer cells, *FERMT2* enhances *TGF-β1* signaling by upregulating *TGFβ-RI*, thereby driving the expression of *FN* and *COL6A1*. *FERMT2* further promotes the secretion of COL6A1-enriched exosomes, which are taken up by GCAFs to reinforce their myofibroblastic phenotype. This, in turn, augments *TGF-β1* signaling within GCAFs, while GCAF-derived TGF-β1 promotes gastric cancer cell migration, invasion, and anoikis resistance. Collectively, our findings delineate a *TGF-β1*/*FERMT2*/*COL6A1* positive feedback loop that sustains reciprocal activation between tumor cells and GCAFs, thereby driving peritoneal metastasis. Targeting this pro-metastatic circuit may provide a novel therapeutic avenue to impede gastric cancer progression.

## Supplementary Material

Supplementary tables.

## Abbreviations

PM: Peritoneal metastasis
GC: Gastric cancer
CAFs: Cancer-associated fibroblasts
GCAFs: Gastric cancer-associated fibroblasts
TGF-β1: Transforming growth factor-beta 1
TME: Tumor microenvironment
TAM: Tumor-associated macrophage
CSF-1: Colony-stimulating factor 1
OSCC: Oral squamous cell carcinoma
EMT: Epithelial-mesenchymal transition
ECM: Extracellular matrix
NFs: Normal fibroblasts
siRNAs: Small interfering RNAs
NC: Negative controls
shRNAs: Short hairpin RNAs
PCA: Principal component analysis
UMAP: Uniform manifold approximation and projection
t-SNE: t-distributed stochastic neighbor embedding
CDS: Cell data set
GSEA: Gene set enrichment analysis
TMAs: Tissue microarrays
IHC: Immunohistochemical
HPA: Human protein atlas
scRNA-seq: Single-cell RNA sequencing
CAF_myo: CAFs with a myofibroblastic phenotype
FN: Fibronectin
CM: Conditioned media
BP: Biological process
TGFβR1: TGF-β receptor 1
miRNAs: MicroRNAs
3'-UTR: 3' untranslated region
MSC: Mesenchymal stem cell
ceRNA: Competitive endogenous RNA
