# Supplementary material for: TGF-βI/FERMT2/COL6A1 Reciprocal Loop Drives Tumor-Stroma Crosstalk and Promotes Peritoneal Metastasis in Gastric Cancer
Source: Int J Biol Sci. 2025 Sep 12;21(13):5859–73. doi: 10.7150/ijbs.119895 (PMC12509910; doi:10.7150/ijbs.119895)
Supplement: Supplementary file 1 — Supplementary tables. [file ijbsv21p5859s1.pdf]

TABLE S1 The primary antibodies applied in this study were shown as follows.

Name	Article numbers (brand)
S100A4	16105-1-AP (Proteintech)
ZEB2	14026-1-AP (Proteintech)
Kindlin 2	11453-1-AP (Proteintech)
Beta Actin	81115-1_RR (Proteintech)
Vimentin	60330-1-Ig(Proteintech)
N-cadherin	122018-1-AP (Proteintech)
Collagen Type VI	17023-1-AP (Proteintech)
CD63	25682-1-AP (Proteintech)
TSG101	28283-1-AP (Proteintech)
Calnexin	10427-2-AP (Proteintech)
TGF beta 1	26155-1-AP (Proteintech)
Fibronectin	66042-1-Ig (Proteintech)
TGF β -RI	30117-1-AP (Proteintech)
FAP	11779-1-AP (Proteintech)
α -Smooth Muscle Actin	14395-1-AP (Proteintech)
SMAD2/3	8685 (CST)
Smad2 (phospho S467)	ab53100 (abcam)
secondary antibodies	AS014 (Abclonal), SA00001-1 (Proteintech)
Fluorescent secondary antibody	SA00013-4, SA00014-10 (Proteintech)

TABLE S2 miR-RNA, siRNA and shRNA sequences in this study were shown as follows.

Name	Sequence (5′ – 3′)
sh-Control	TTCTCCGAACGTGTCACGT
sh-FERMT2-1	CCGAAGAACTTTCTCTCTTAA
sh-FERMT2-2	GCGGACAGTTCTTACAACTTA
siFERMT2-1	CCUUGCUGCUCCGAUUCAA (dT)(dT)
siFERMT2-2	GCCCAGGACUGUAUAGUAA (dT)(dT)
siFERMT2-3	GCUAGAUGACCAGUCUGAA (dT)(dT)
siSmad2-1 (5'-3')	GAAGAGGAGUGCGCUUAUA(dT)(dT)
siSmad2-2 (5'-3')	GAGUACACCAAAUACGAUA(dT)(dT)
siSmad2-3 (5'-3')	CGUCUAUCAGCUAACUAGA(dT)(dT)
siCOL6A1-1 (5'-3')	CCAAGCGCUUCAUCGACAA(dT)(dT)
siCOL6A1-2 (5'-3')	GGUCAAGGAGAACUAUGCA(dT)(dT)
siCOL6A1-3 (5'-3')	CGAUAACAACGACAUUGCA(dT)(dT)
siZEB2-1	GACGAUAGUUCGGAGGAUG(dT)(dT)
siZEB2-2	GGUACAGUGAAGAAUGCAA(dT)(dT)
siZEB2-3	GGAGUUACUUCUCCUAAUA(dT)(dT)
si-Control	UUCUCCGAACGUGUCACGU(dT)(dT)
miR-138 mimics	AGCUGGUGUUGUGAAUCAGGCCG
miR-200a-5p- mimics	CAUCUUACCGGACAGUGCUGGA
NC-mimics	UCACAACCUCCUAGAAAGAGUAGA
